# Synthesis of Fluorescent Core-Shell Metal Nanohybrids: A Versatile Approach

**DOI:** 10.3390/ma9120997

**Published:** 2016-12-09

**Authors:** Marina Alloisio, Melania Rusu, Stefano Ottonello, Massimo Ottonelli, Sergio Thea, Davide Comoretto

**Affiliations:** Dipartimento di Chimica e Chimica Industriale (DCCI), Università di Genova, Via Dodecaneso 31, 16146 Genova, Italy; r.melania@virgilio.it (M.R.); stefano.ottonello@live.it (S.O.); massimo.ottonelli@unige.it (M.O.); sergio.thea@unige.it (S.T.)

**Keywords:** noble metal nanoparticles, core-shell architecture, self-assembly, spectroscopic properties, polymeric matrix

## Abstract

A flexible way of fabricating core-shell noble metal-organic nanohybrids with tailored chemical and spectroscopic properties is proposed here. The synthetic protocol consists of a multi-step procedure able to guarantee acceptable reproducibility of core size and shape as well as control of the organic outer layer. The proposed method highlights limitations in obtaining highly controllable products, although the heterogeneity degree of the nanostructures is in line with that expected from bottom-up approaches in solution. Selective functionalization of the nanohybrids with properly-substituted fluorescent dyes under variable experimental conditions allowed the preparation of composite systems of tunable spectroscopic properties to be employed as nanoprobes in sensing or photonic applications. To this end, preliminary investigation on embedding the nanohybrids in compatible polymeric matrices is also reported.

## 1. Introduction

Core-shell metal nanohybrids (NHs) are a class of compounds of particular interest, consisting of a metallic nucleus protected by organic molecules, which are selectively anchored on the core surface to combine the typical properties of metal nanostructures with the chemical-physical characteristics of the adsorbates. In addition to its capability of passivating metal surfaces, the organic shell is a suitable support for a multitude of functional groups, chromophores, and biomolecules. As a consequence, the resulting material provides, in terms of properties and distinctive features, optimized returns with respect to the single components, thus finding application in various fields including photonics and sensing [1,2,3,4,5].

Among these systems, those based on noble metals, such as gold and silver, seem to be the most promising candidates for the development of optical probes, thanks to their intense absorptions over a large spectral range from the visible to the near infrared (NIR) [6,7]. These absorptions, commonly defined as surface plasmon resonance (SPR), are generated by the collective oscillation of conduction band electrons in the presence of an electromagnetic field. In the case of gold and silver nanoparticles (AuNPs and AgNPs, respectively), the SPR bands have extinction cross sections about two order of magnitude larger than ordinary molecular chromophores and are more intense than those of other metal nanoparticles (MNPs), due to the weak coupling of inter-band transitions. In addition, the spectroscopic properties of nanosized gold and silver depend on the structural parameters of the metal colloids, such as shape, size, and state of aggregation [8], but are also strongly influenced by the chemical nature of the outer layer and can be properly implemented by derivatization with accessory chromophores or fluorophores [9].

In particular, attractive changes can be observed on metal nanostructures coated with emitting moieties, related to the rate of excitation, the value of quantum yield (Φ) or fluorescence lifetime (τ), and the photostability. Different parameters play a role in the interaction mechanism between the metal core and the fluorophores, such as the nanoparticle size and shape [10,11] or the distance between the photoactive species and the metal surface [12]. Chromophores positioned within ca. 5 nm from the nanoparticle interact electronically with its surface by quenching the fluorescence through electron and energy transfer processes, which involve surface energy transfer from the dye molecules [13]. Total quenching of the singlet excited state is observed when fluorophores are densely packed on the metal surface, thus attracting attention for biomedical applications [14], such as the development of novel diagnostics devices for the identification of molecules present in very low concentrations (<50 nmol/L) [13]. On the contrary, fluorophores positioned at ca. 10 nm enhance their fluorescence up to 100-fold [11,15,16]. This effect, commonly known as metal enhanced fluorescence (MEF), is also of great importance for the development of fluorescence-based techniques [17]. Moreover, metal nanoparticles were proved to stabilize nearby fluorophores against photo-bleaching, thus facilitating their use in bio-imaging [18].

In gold nanoparticles linked to fluorophores, the emission can be quenched by fluorescence resonance energy transfer (FRET) [19], photoinduced electron transfer (PET) [20] or nanosurface energy transfer (NSET) [21] pathways. To date, all these mechanisms have been successfully exploited for applications in biology and medicine [22,23]. Recently, visualization methods employing AuNPs in optical microscopy, in particular confocal laser microscopy, have been on the rise [24]. In addition to their fluorescence property, AuNPs can enhance Raman scattering for the development of surface enhanced Raman spectroscopy (SERS)-based sensing [25], in which the intense optical frequency field originating from the plasmon resonance of AuNPs can be used for single molecule detection [26].

Although silver exhibits many advantages over gold, such as higher extinction coefficient, sharper extinction band, higher scattering-to-extinction ratio, and extremely high field enhancement, it has been employed far less in sensing devices, with the exception of sensors based on surface enhanced spectroscopies [27]. The reason for its limited use is mainly due to the lower chemical stability of silver nanoparticles when compared to gold counterparts. Nevertheless, recent studies have been focused on means of protecting AgNPs that significantly improve their chemical stability. As a consequence, silver nanoparticles are rapidly gaining in popularity and alternative strategies for the development of optical sensors and imaging labels based on the uncommon optical properties of these metal nanostructures are being explored. AgNPs have also demonstrated their potential in label-free bioassays, in MEF-based sensors [28,29] or cell imaging applications [30].

**Scheme 1:**
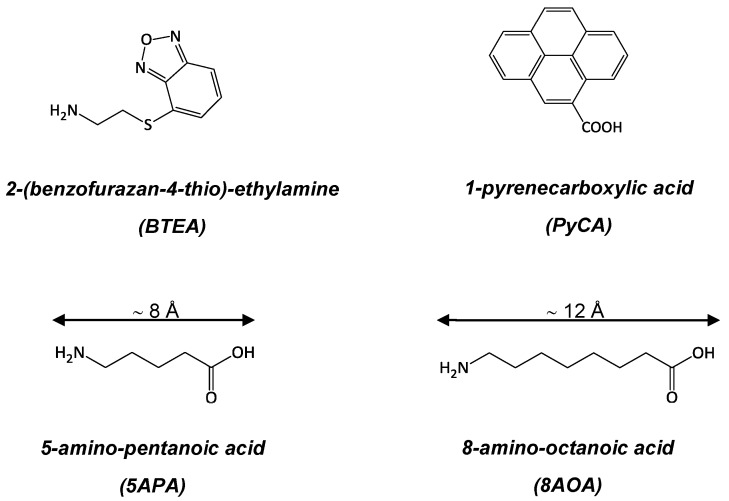
Chemical formulas of the fluorescent probes (**top**) and alkyl spacers (**bottom**) employed in the synthesis of the core-shell metal nanohybrids.

Starting from these premises, this work was focused on the design and preparation of novel core-shell nanohybrids with tailored optical properties to be integrated into conventional devices as well as into innovative systems such as one-dimensional planar polymeric photonic crystals (1D-PhC) [31]. The novel composite materials were obtained by assembling at the nanoscale selected fluorophores on gold and silver nanoparticles of nearly controlled size and shape. In detail, two fluorescent probes carrying different end-groups were tested, the amino-substituted benzofurazan BTEA (2-(benzofurazan-4-thio)-ethylamine) and the carboxylic-functionalized pyrene PyCA (1-pyrenecarboxylic acid), the chemical formulae of which are reported in Scheme 1. The pyrene compound is a commercial product, whereas the benzofurazan derivative was synthesized in our laboratories [32].

In order to investigate the effect of the metal-fluorophore distance on the degree of dye covering as well as on the emitting properties of the nanohybrids, the fluorescent moieties were anchored to the core surface through spacers of different length, 5APA (5-amino-pentanoic acid) and 8AOA (8-amino-octanoic acid) (Scheme 1). Different synthetic ways were explored to find the best performing protocols in terms of reproducibility and optimized properties of the resulting products. Wet methods were empowered to comply with the new directives regarding environmental impact. An estimation of the nanostructures size and shape from their UV-vis spectra based on the fitting model proposed by Amendola and Meneghetti [33] is also reported.

## 2. Results and Discussion

Core-shell metal nanohybrids (NHs) were obtained through a wet, flexible multi-step procedure, briefly summarized below. First, silver and gold nanoparticles were synthesized by reducing their corresponding salts in water, either in the presence or in the absence of stabilizing agents. Then, AgNPs and AuNPs were used as templates for the passivation with spacers and dyes through a combined protocol of ligand-exchange, self-assembly, and functionalization reactions. Once formed, the products were fractioned by centrifugation in order to obtain colloids of selected size and shape. In the third step, crude nanohybrids were purified from unreacted species by means of ultrafiltration with the dual purpose of obtaining clean products and evaluating the covering degree of the photoactive species on the metal cores. Finally, the purified nanohybrids were incorporated into a polymeric matrix with the aim of testing their potentialities in the fabrication of all-polymer photonic devices. To this end, different water-soluble polymers were exploited under different experimental conditions, such as the polysaccharide chitosan (H-Chit) or the synthetic polyvinyl alcohol (PVAL).

All preparation steps, described in detail in the Experimental Section, were monitored by spectroscopic investigation. The obtained results are shown in the following sections together with the morphological characterization carried out on some samples.

### 2.1. Passivation of Noble Metal Nanoparticles with Amino-Carboxylic Spacers

Pre-synthesized gold and silver nanoparticles were protected against coagulation and/or agglomeration with the amino-carboxylic spacers 5APA and 8AOA by taking advantage of the well-known capability of noble metal surfaces to spontaneously assemble properly-functionalized hydrocarbon chains. Two different templates were tested for the gold sample, namely the 1-propylamine-capped gold nanoparticles PA@AuNPs and the “bare”, freshly-prepared gold nanoparticles b-AuNPs, whereas only “unprotected” b-AgNPs were employed for the silver counterparts.

**Figure 1:**
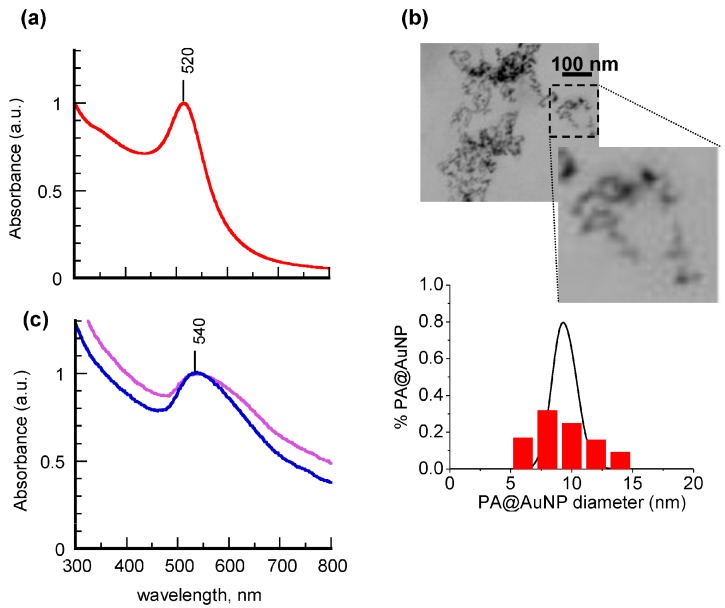
(**a**) Experimental absorption spectrum of an aqueous suspension of 1-propylamine-capped gold nanoparticles PA@AuNPs (solid red line); (**b**) Scanning transmission electron microscopy (STEM) images of PA@AuNPs at two different magnification orders and the corresponding dimensional histogram (red bars) with the log-normal size distribution (LNMG, black line) obtained from the Mie and Gans (MG) fitting model; (**c**) Absorption spectra of aqueous suspensions of 5APA@AuNPs (purple line) and 8AOA@AuNPs (blue line); the absorption intensities are normalized at the absorbance value of the plasmon wavelength. Fitting parameters: for LNMG line: average diameter = 7.4 nm with σ = 0.1 nm; 96% spherical particles and 4% elongated particles with average aspect ratio = 2.7.

Figure 1 shows the absorption spectra recorded from an aqueous suspension of PA@AuNPs before and after chemisorption of 5APA or 8AOA molecules on the gold cores; to facilitate the comparison of spectra, the corresponding intensities were normalized at the maximum wavelength.

The spectrum of the propylamine derivative (Figure 1a, solid line) is dominated by the sharp, well-defined band centered at 520 nm, typical of gold nanoparticles. The fitting of the spectral profile through a program based on the Mie and Gans (MG) model [33] gave a calculated extinction profile (Figure 1a, dotted line) with an accuracy of about 98%. The fitting parameters, obtained from a population of 96% spherical particles and 4% elongated particles (aspect ratio around 2.7) with 3.7-nm average radius, are in good agreement with the STEM (scanning transmission electron microscopy) images of Figure 1b, which reveal the presence of quite homogeneous particles of 9.5 ± 3.5 nm diameter and nearly spherical shape (aspect ratio = 1.2 ± 0.2). Also the log-normal size distribution (LNMG) of the fitting model (Figure 1b, black line) is shown to estimate quite well the STEM measured experimental size histogram (Figure 1b, red bars). After having tested the reliability of the theoretical approach on PA@AuNPs, the MG method was systematically applied in order to gain morphological information on the nanohybrids from their absorption spectral profiles.

The spectra of 5APA@AuNPs and 8AOA@AuNPs, reported in Figure 1c, are characterized by a significant broadening and a 50-nm red-shift of the corresponding plasmon bands with respect to their precursor PA@AuNPs. These features can be associated with changes of metal core morphology, induced by the ligand-exchange reaction (LER), although the technique was proved to be a versatile tool to introduce novel chemical functionalities on the AuNPs monolayer in a divergent fashion [34]. The MG method estimated a remarkable increase of the samples heterogeneity following the amino-carboxylic acids anchoring with the growth of the elongated particles up to 67%. More details on the fitting parameters are provided in Supplementary Materials (Figure S1).

**Figure 2:**
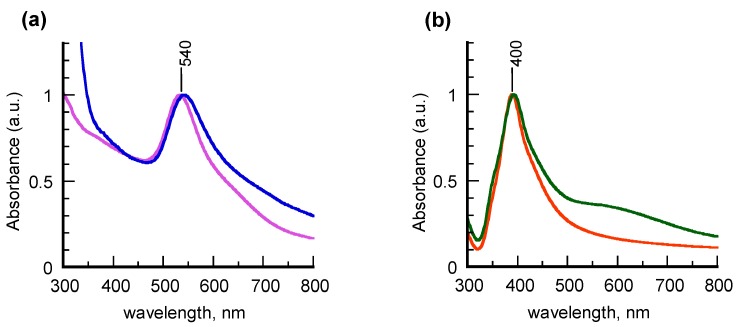
(**a**) Absorption spectra of aqueous suspensions of 5APA@AuNPs (purple line) and 8AOA@AuNPs (blue line) obtained from “naked” AuNPs; the absorption intensities are normalized at the absorbance value of the plasmon wavelength; (**b**) Absorption spectra of aqueous suspensions of 5APA@AgNPs (orange line) and 8AOA@AgNPs (green line) obtained from “naked” AgNPs; the absorption intensities are normalized at the absorbance value of the plasmon wavelength.

Different results were obtained from chemisorption of 5APA and 8AOA on the “unprotected” colloids b-AuNPs and b-AgNPs, as displayed in the UV-vis spectra of Figure 2. Well-defined SPR bands centered at 540 and 400 nm were found for gold and silver colloids, respectively, to indicate that in these samples the geometry of the metal cores is not significantly altered by the adsorbate self-assembly. According to the MG fitting method, the spectral features are consistent with formation of nearly-spherical 8-nm AuNPs and 7-nm AgNPs (Figure S2 in Supplementary Materials).

**Scheme 2:**
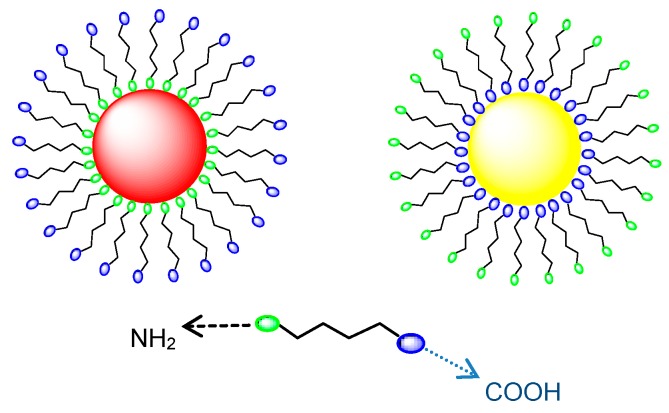
Sketched representation of the core-shell architecture of 5APA@AuNPs (on the **left**) and 5APA@AgNPs (on the **right**).

The employment of these two spacers of different lengths induces insignificant modifications in the absorption lineshapes, which allows the different water solubilities of the protecting agents and even-odd chain effects on the chemisorption process to be neglected. Nevertheless, owing to the well-documented, different affinity of carboxylic functionalities for gold and silver surfaces [35,36,37], 5APA and 8AOA molecules are supposed to arrange with opposite orientation on AuNPs and AgNPs. As sketched in Scheme 2, anchoring on gold colloids is carried out by the amino groups, so allowing the passivated nanostructures to exhibit surface carboxylic substituents. Conversely, the spacer anchoring on the Ag surface occurs in the opposite way and, consequently, amino-substituents are freely exposed to the outside in the silver counterparts. To prove this assumption, the nanohybrids were selectively reacted with differently-functionalized fluorescent probes, the amino-endowed benzofurazan BTEA (2-(benzofurazan-4-thio)-ethylamine) and the carboxylic-derivatized pyrene PyCA (1-pyrenecarboxylic acid), as explained in the following section.

### 2.2. Tailored Functionalization of Noble Metal Nanohybrids with Fluorophores

**Figure 3:**
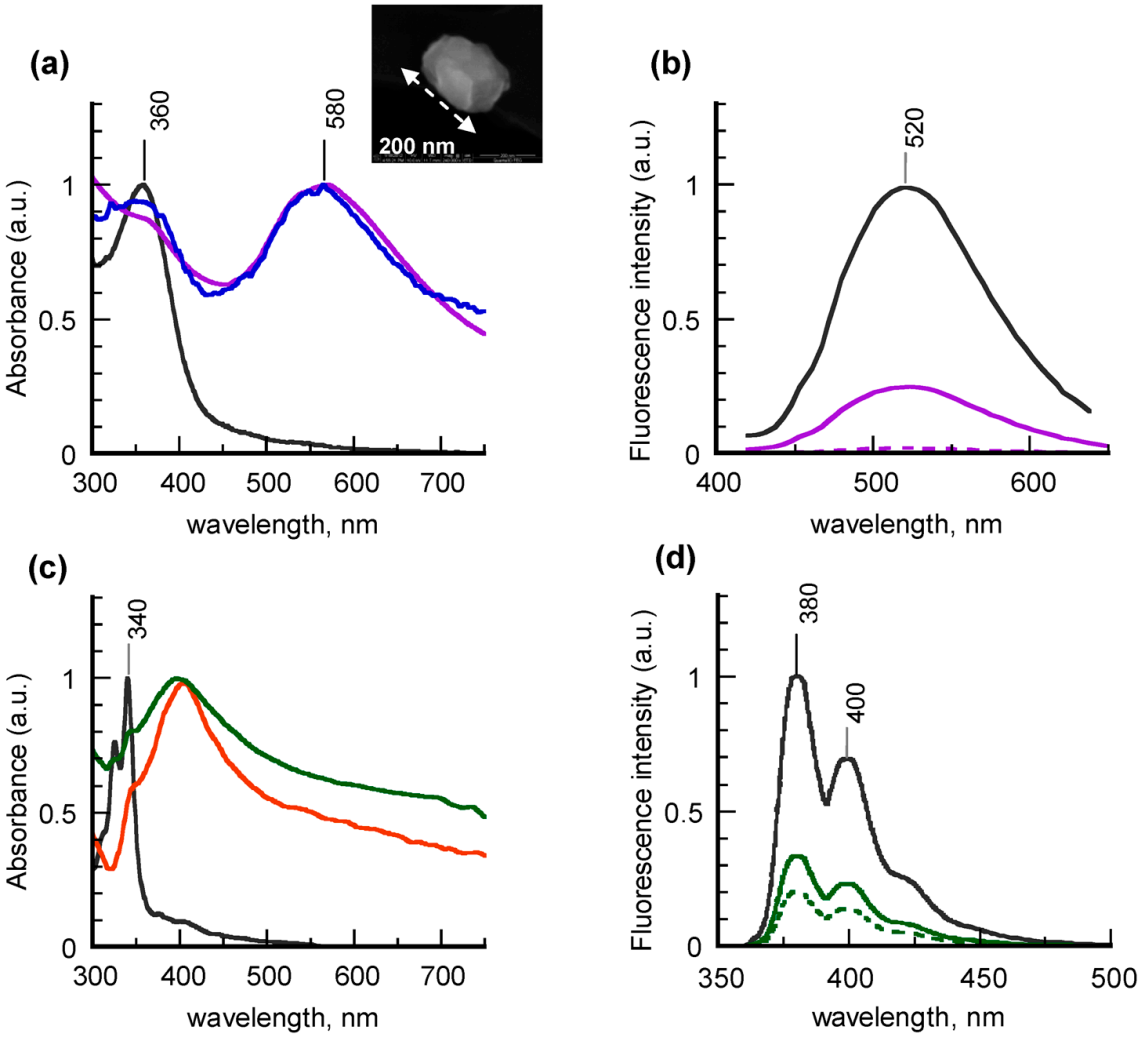
(**a**) Absorption spectra of aqueous suspensions of BTEA-5APA@AuNPs (purple line), and BTEA-8AOA@AuNPs (blue line) compared with the absorption spectrum of free BTEA (2-(benzofurazan-4-thio)-ethylamine) in water (grey line); the intensities are normalized at the absorption maximum value. STEM image of a typical larger nanoparticle of BTEA-5APA@AuNPs is reported alongside; (**b**) Fluorescence spectra of aqueous suspensions of BTEA-5APA@AuNPs with 5% (purple solid line) and 18% (purple dashed line) dye covering, compared with the fluorescence spectrum of free BTEA in water (grey solid line); the fluorescence spectra, recorded by exciting at 334-nm wavelength, are normalized at the same value of BTEA concentration; (**c**) Absorption spectra of aqueous suspensions of PyCA-5APA@AgNPs (orange line), and PyCA-8AOA@AgNPs (green line) compared with the absorption spectrum of free PyCA (1-pyrenecarboxylic acid) in water (grey solid line); the intensities are normalized at the absorption maximum value; (**d**) Fluorescence spectra of aqueous suspensions of PyCA-8AOA@AgNPs with 5% (green solid line) and 11% (green dashed line) dye covering, compared with the fluorescence spectrum of free PyCA in water (grey solid line); the fluorescence spectra, recorded by exciting at 334-nm wavelength, are normalized at the same value of PyCA concentration.

BTEA molecules were linked to 5APA@AuNPs and 8AOA@AuNPs through a classic carbodiimide-assisted condensation, carried out at variable ratio of reagents by following the protocol described in Section 3.5. In all cases, stable products were obtained, the spectroscopic characterization of which is reported in Figure 3a together with that of the free fluorophore in water.

Typical electronic absorption spectra of the nanohybrids show two bands, the higher-energy one assigned to the dye and the second one, centered at 580 nm, corresponding to the gold SPR. Notice, however, that while the contribution of anchored BTEA is unchanged, the plasmon band is nearly 40-nm red-shifted with respect to the “naked” gold precursors. This evidence is due to the partial coagulation of the metal cores induced by the functionalization reaction, as confirmed by the STEM image of Figure 3a, which reveals the presence of gold clusters up to 200-nm size. UV-vis investigation carried out on 5APA@AuNPs coupled with 1-(3-dimethylaminopropyl)-3-ethylcarbodiimide hydrochloride (EDC) at different reaction times confirmed that the cores coagulation happens during the activation process of the surface carboxylic groups with the carbodiimide (Figure S3 in Supplementary Materials). Apart from the SPR shift, the nanohybrid spectra are very similar to those of the single photoactive moieties, thus indicating that no significant interaction was established between the two components at the ground state.

Spectroscopic characterization of the wastewaters collected during the purification process of the crude products by ultrafiltration technique allowed quantitative determination of the anchored dye percentage, which turned out to vary from 5% to 18% in terms of dye loading efficiency [38], depending on the experimental conditions adopted. These values are consistent with the dye contributions to the absorption profiles, as revealed by the corresponding deconvolution spectra (Figure S4 in Supplementary Materials).

Much more evident are the changes caused by the chemisorption on the emitting properties of BTEA, as shown in the fluorescence spectra (Figure 3b), corresponding to BTEA-5APA@AuNPs at increasing dye covering and to the free dye in water. For the sake of comparison, the emission intensities were normalized at the same value of fluorophore concentration. It is evident that the photoluminescence profile of the functionalized nanohybrids is the same of the unbound dye, with the maximum picked around 520 nm. However, the fluorescence intensities strongly decrease with increasing the BTEA covering, most probably because of both quenching and self-absorption phenomena [25]. In particular, by raising the dye covering degree up to nearly 4-fold value, the fluorescence is almost completely quenched. The results did not remarkably change with the type of spacers, which suggests that the nanohybrids emission is not significantly influenced by the type of spacers, at least in the range of chain lengths explored.

Also PyCA-functionalized silver nanoparticles were obtained by reacting 5APA@AgNPs and 8AOA@AgNPs with PyCA molecules in the presence of EDC as coupling agent, as described in detail in Section 3.6. Typical examples of absorption and fluorescence spectra obtained from colloidal aqueous suspensions and free dye in water are compared in Figure 3c. Once again, the UV-vis spectra of the nanohybrids look very similar to the sum of those of the single components, with the doublet under 350 nm attributable to the pyrene ring and the well-resolved silver SPR peaked at 400 nm, to confirm that in this type of core-shell nanohybrids no interactions at the ground level are established between the composing moieties. Notice that, unlike the previous specimens, in silver samples the plasmon position is not affected by the linking with the dye. This result can be explained by taking into account that the carbodiimide activation, being devoted to the carboxylic functionalities, in this case is directed to the fluorophore molecules and not to the spacer-passivated colloids. Quantitative determination of the linked dye, calculated as explained before, gave percentage values of the dye loading efficiency ranging from 5% to 11%, depending on the experimental conditions adopted. In general PyAC-decorated colloids showed a dye-covering density lower with respect to their gold counterparts, most probably due to the higher steric hindrance of the pyrene rings.

As far as the fluorescence properties are concerned, the fluorescence spectra of PyCA-8AOA@AgNPs at different dye covering, reported in Figure 3d, are not significantly modified in lineshape and position with respect to that of the unbound dye, characterized by the doublet at 380 and 400 nm and by the typical mirror-like features with respect to the absorption profile. Once again the corresponding intensities show a drop as the greater is the percentage of anchored PyCA, although less dramatically with respect to the gold systems.

On the whole, selective anchoring of amino-terminated BTEA molecules on gold nanoparticles and carboxylic-substituted PyCA on silver nanoparticles confirm the different supramolecular architecture of the two core-shell colloids, reported in Scheme 2, with the opposite arrangement of the bi-functionalized alkyl spacers.

Fluorescence quenching was found in this new class of hybrid nanosystems, which turned out to be more relevant for the benzofurazan-gold derivatives. This result was expected on the basis of literature data concerning polycyclic aromatic dyes anchored on metal cores through spacers of length comparable to 5APA and 8AOA [12,13,14]. Moreover, the fluorescence properties of these samples were proved to be tunable by a proper choice of synthetic conditions, which opens the way to their potential use as unlabeled building blocks in sensing and/or optical platforms.

### 2.3. Nanohybrids Embedding in Polymeric Matrix

In order to extend the application range of the new core-shell noble metal nanohybrids beyond the areas that predict use of colloidal suspensions or powders, we investigated their embedding in compatible polymeric matrices with the aim of obtaining films of good optical quality to be integrated in conventional or innovative all-polymer photonic devices.

Preliminary results obtained by incorporating PyCA-decorated silver nanohybrids or spacer-passivated gold nanoparticles in PVAL or chitosan matrix are here reported. The choice of the colloids was dictated by the need to investigate fillers of comparable size to avoid segregation phenomena. Consequently, BTEA-5APA@AuNPs and BTEA-8AOA@AuNPs were not taken into account because of their increased dimensions with respect to the metal precursors.

Homogeneous mixtures were obtained by adding the nanostructures to aqueous solutions of the polymers, to verify the good grade of nanoparticle-polymer compatibility at the liquid state. The colloidal suspensions were proved to be stable against aging and variable experimental conditions, such as acid or alcoholic solutions, thanks to the optimized passivation of the metal cores with the alkylic spacers. The mixtures were then filmed on microscope slides by a dipping technique, as described in detail in Section 3.7.

**Figure 4:**
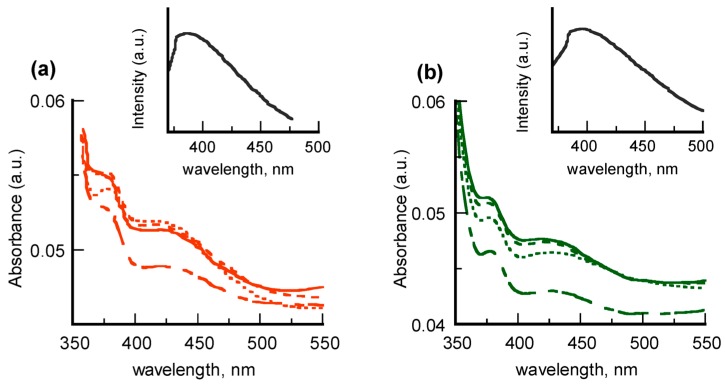
(**a**) Absorption spectra of multilayered films of PyCA-5APA@AgNPs embedded in polyvinyl alcohol (PVAL) matrix, obtained by dipping technique: number of layers = 2 (dashed-dotted line), 5 (dotted line), 9 (dashed line), 12 (solid line); inset: fluorescence spectrum of the bilayer (*n* = 2) film of PyCA-5APA@AgNPs embedded in PVAL matrix obtained by exciting at 334-nm wavelength; (**b**) Absorption spectra of multilayered films of PyCA-8AOA@AgNPs embedded in PVAL matrix, obtained by dipping technique; number of layers = 2 (dashed-dotted line), 5 (dotted line), 10 (dashed line), 12 (solid line); inset: fluorescence spectrum of the bilayer (*n* = 2) film of PyCA-8AOA@AgNPs embedded in PVAL matrix obtained by exciting at 334-nm wavelength.

Spectroscopic investigation of multilayered films of PVAL with incorporated PyCA-5APA@AgNPs and PyCA-8AOA@AgNPs is reported in Figure 4.

Absorption features confirming the presence of dispersed colloids in the transparent polymeric matrix are found in the UV-vis profiles (left side). The SPR bands of nanostructured silver are evident at 450 nm, whereas the absorptions around 350 nm, although poorly defined, can be attributed to the pyrene rings. Fluorescence spectra reported in the insets of Figure 4 (right side) are reminiscent of those of PyCA in water (cf. Figure 3b), thus confirming this hypothesis. Nevertheless, changes in the spectral lineshape and position are evident in both absorption and fluorescence spectra. By taking into account the tight dependence on chemical environment of the silver plasmonic band as well as of the pyrene emitting properties [39], this evidence is most probably related to local dielectric changes rather than to nanoparticles aggregation and/or coagulation in the polymeric matrix. The trend of the absorption intensities, which grow to 5-layered films and then saturate, indicates the presence of washout effects in the subsequent dipping depositions. Insignificant behavior differences were detected in the colloids coated with 5APA or 8AOA, to underline that the spacer length did not affect the dispersion within the PVAL matrix.

**Figure 5:**
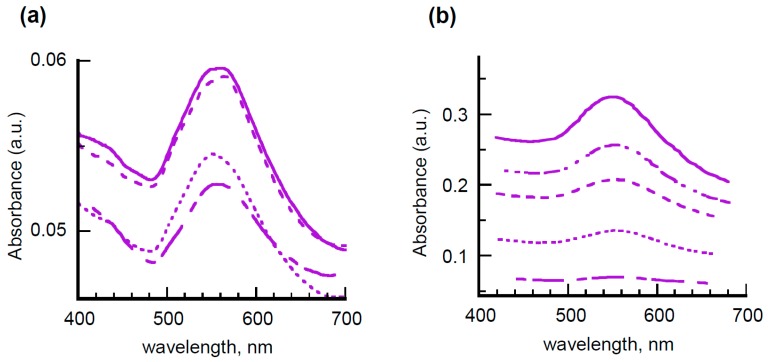
(**a**) Absorption spectra of multilayered films of 5APA@AuNPs embedded in PVAL matrix, obtained by dipping technique; number of layers = 2 (dashed-dotted), 4 (dotted line), 6 (dashed line), 8 (solid line); (**b**) Absorption spectra of multilayered films of 5APA@AuNPs embedded in chitosan matrix, obtained by alternate dipping with a hyaluronate matrix; number of layers = 2 (dashed-dotted line), 5 (dotted line), 8 (dashed line), 10 (double-dashed line), 12 (solid line).

Very similar results were obtained by incorporating passivated gold colloids in the same polymeric matrix, as shown by the UV-vis spectra of multilayered films filled with 5APA@AuNPs of Figure 5a, thus evidencing that the film homogeneity is not affected by the chemical properties of the nanofillers but rather by the technique chosen for the layer deposition.

To prove this point and with the aim of better controlling the film thickness, another type of polymeric matrix was tested and the dipping procedure modified consequently. Based on the widespread use of amino-derivatives for the functionalization of glass substrates [40] and the proven ability of chitosan to stabilize gold and silver nanoparticles [41,42], high molecular mass chitosan (H-Chit) was selected as a dispersant matrix, assuming that the polysaccharide, thanks to its amino substituents, could be anchored more effectively to the microscope slide, thus ensuring the formation of more uniform layers. To further increase the homogeneity of the final multilayer, the deposition of H-Chit mixed with nanohybrids was alternated with that of hyaluronic acid (HyA), an anionic polysaccharide supposed to establish surface electrostatic interactions with the chitosan layer through its side carboxyl groups, thus operating as an insulating layer against washout and water percolation effects. Figure 5b reports the spectra of films prepared by subsequent deposition of bilayer of chitosan filled with 5APA@AuNPs and HyA. It is evident that the gold SPR band, centered around 550 nm, constantly increases on increasing the number of bilayers together with the spectral background, owing to scattering or reflection losses. These features are compatible with the presence of bilayers of quite uniform thickness, unlike the previous cases. Once again, insignificant behavior differences were found with 8AOA@AuNPs incorporated in both PVAL and H-Chit matrices, as displayed in Figure S5 of Supplementary Materials.

Summarizing the results obtained, it was demonstrated that the new class of nanohybrids can be successfully embedded in water-soluble polymeric matrices without losing their individuality as primary particles. The composite mixtures tested so far turned out to be filmable, but the homogeneity of the final films mainly depends on the deposition technique adopted and needs to be significantly improved to reach the uniformity degree required by technological applications.

## 3. Materials and Methods

### 3.1. Chemicals

Reagents and solvents employed were spectroscopic grade commercial products and were used as received. High molecular mass chitosan (H-Chit, acetylation degree = 22.8%, Mw = 1.34 × 10^6^ g/mol in terms of repeating units) was purchased from Fluka. Aqueous solutions were made with ultra-high-purity water, twice distilled prior to use. Hyaluronic acid (HyA) in form of sodium salt was produced by Pharma Cosmetics Polli (Milano, Italy) without producing technical specifications. Polyvinyl alcohol (PVAL), hydrolyzed up to 87%–89% (Mw = 85,000–124,000 g/mol), was a Sigma-Aldrich Italia (Milano, Italy) product.

The glassware was thoroughly cleaned by using a fresh “piranha” solution, prepared by mixing hydrogen peroxide (35% *v*/*v*) and concentrated sulfuric acid in the ratio of 1:3 (*v*/*v*) at room temperature for 3 h. When the treatment time was over, the glassware was washed with bi-distilled water for immediate use.

### 3.2. Synthesis of Propylamine-Protected Gold Nanoparticles (PA@AuNPs)

In a flask containing 100 mL of bi-distilled water and equipped with a magnetic bar, 1 mL of HAuCl_4_ aqueous solution (25 mmol/L), 0.2 mL of 1-propylamine (PA) in water (25 mmol/L), and 1 mL of a freshly prepared solution of NaBH_4_ (25 mmol/L) were added under constant stirring. The mixture color immediately turned from orange to red-purple because of the formation of gold nanoparticles coated with PA molecules. The crude sample was stirred overnight at room temperature to ensure full reaction and then centrifuged at 14,500 rpm for 15 min. The supernatant fraction, corresponding to about 50% of the total gold amount, was collected and used for passivation with 5APA and 8AOA spacers without further purification from free PA molecules.

### 3.3. Synthesis of “Bare” Gold and Silver Nanoparticles (b-AuNPs and b-AgNPs)

In a volumetric flask equipped with a magnetic bar, 40 mL of AuHCl_4_ or AgNO_3_ aqueous solution (2 mmol/L) were diluted with 20 mL of bi-distilled water and added of 40 mL of a freshly-prepared solution of NaBH_4_ (4 mmol/L) under vigorous stirring. The suspensions, which immediately turned red or yellow, respectively, were stirred for 30 min at room temperature and then immediately used for passivation with 5APA and 8AOA spacers without size fractioning to avoid coagulation and/or aggregation of the metal cores.

### 3.4. Passivation of Gold and Silver Nanoparticles with 5APA and 8AOA Spacers

Chemisorption of 5APA and 8AOA on gold and silver nanoparticles was carried out by incubating the colloidal suspensions with an aqueous solution of the alkyl spacers. In a typical protocol, 10 mL of aqueous suspensions of gold and silver nanoparticles, previously synthesized as described in Section 3.2 and Section 3.3, were added with proper aliquots of 5APA or 8AOA in water to get metal:spacer ratios ranging from 1 to 5. The mixture was left at room temperature under stirring for 48 h to complete the self-assembly on the metal surfaces. When the reaction time was over, the samples obtained from PA@AuNPs were characterized without any purification treatment. Samples obtained from b-AuNPs and b-AgNPs were instead subjected to centrifugation at 14,500 rpm for 15 min to separate the supernatant fraction, containing nanoparticles more uniform in size and shape.

### 3.5. Synthesis of BTEA-Functionalized Nanohybrids (BTEA-5APA@AuNPs and BTEA-8AOA@AuNPs)

A 0.05 mol/L aqueous solution of the coupling agent EDC (1-(3-dimethylaminopropyl)-3-ethylcarbodiimide hydrochloride) was added to a proper aliquot of 5APA@AuNPs or 8AOA@AuNPs to get a spacer:EDC ratio = 1:1. The so-obtained suspensions were allowed to gently stir for 30 min at room temperature. Then a fixed aliquot of BTEA in water was added to the nanoparticles suspensions to obtain an EDC:fluorophore ratio ranging from 1:1 to 3:1 and stirred at room temperature overnight. The BTEA-derivatized gold nanoparticles were then separated from the excess dye by ultracentrifugation in an Amicon cell, Mod. 8200. In this way, 10 mL of a crude suspension was introduced into the cell containing an ultrafiltration membrane of regenerated cellulose of 10,000 NMWL (nominal molecular weight limit) and washed with 10 mL of bi-distilled water three times. The wastewaters, composed by aqueous solutions of free BTEA molecules, were collected and spectroscopically analyzed to calculate the solute concentration by means of the fluorophore titration curves (Figure S6 in Supplementary Materials).

### 3.6. Synthesis of PyCA-Functionalized Nanohybrids (PyCA-5APA@AgNPs and PyCA-8AOA@AgNPs)

A 0.05 mol/L aqueous solution of the coupling agent EDC (1-(3-dimethylaminopropyl)-3-ethylcarbodiimide hydrochloride) was added to a proper aliquot of PyCA in water to get a PyCA:EDC ratio = 1:1 and left to gently stir for 30 min at room temperature. Then, a fixed aliquot of the so-obtained solution was added to the suspensions of 5AVA@AgNPs or 8AOA@AgNPs to obtain a spacer: fluorophore ratio ranging from 1:1 to 3:1 and stirred at room temperature overnight. The PyCA-derivatized silver nanoparticles were separated from the excess dye by ultracentrifugation following the procedure described in Section 3.5. Also in this case the wastewaters, composed of aqueous solutions of free PyCA molecules, were collected and spectroscopically analyzed to calculate their concentration by means of the fluorophore titration curves (Figure S7 in Supplementary Materials).

### 3.7. Preparation of Polymeric Film Embedding Nanohybrids

Multilayered films of PVAL containing PyCA-functionalized silver nanoparticles or spacer-passivated gold nanoparticles were prepared through subsequent depositions on microscope slides of the corresponding hydro-alcoholic (50% *v/v*) suspensions by a dipping technique. In detail, 60 mL of a PVAL solution, obtained by dissolving 2 g of polymer in 100 mL of a H_2_O:EtOH mixture (50% *v/v*), was added of a 40 mL-aliquot of the nanoparticle aqueous suspensions and left under constant stirring at room temperature for 3 h. Then, a microscope slide was dipped for 1 min into the hydro-alcoholic suspension, extracted at approximately 3 mm/s rate and dried in a nitrogen flow. The deposition procedure was repeated several times in order to prepare polymeric films composed of 2–12 layers.

Films composed by alternating layers of H-Chit and HyA with embedded spacer-passivated gold nanoparticles were prepared through subsequent depositions on microscope slides of the corresponding aqueous suspensions by dipping technique. In detail, 60 mL of a H-Chit solution, obtained by dissolving 2 g of polymer in 100 mL of an aqueous solution of CH_3_COOH (2% *v*/*v*), was added to a 40 mL-aliquot of the nanoparticle aqueous suspensions under constant stirring at room temperature for 3 h. Then, a microscope slide was dipped for 1 min into the nanoparticle-containing H-Chit suspension, slowly extracted and dried in a nitrogen flow. Once dried, the sample was dipped for 1 min into a 2% (*w*/*v*) aqueous solution of HyA, slowly extracted and dried in a nitrogen flow to obtain the first AuNP-HChit/HyA bilayer. The two-step procedure was repeated several times in order to prepare polymeric films composed of 1–7 bilayers.

### 3.8. Instrumentation

*UV-vis spectroscopy*. Electronic absorption spectra were recorded at room temperature on a Perkin-Elmer Lambda 9 spectrophotometer using fused silica cuvettes of different path lengths.

*Fluorescence spectroscopy.* Steady state fluorescence spectra were measured by using a Perkin Elmer MPF-44A spectrofluorimeter with excitation at 334 nm using fused silica cuvettes of different path length. Emission intensities were normalized at a constant value of source intensity by employing a standard of Rhodamine B embedded in a poly(methylmethacrylate) (PMMA) matrix.

*STEM.* The gold nanoparticles were analyzed with a FEI environmental scanning electron microscope (E-SEM) with Field Emission Gun Quanta3D™ and Scanning Transmission Electron Microscope detector, working at 30 kV column bias.

*Ultrafiltration.* Ultrafiltration cell Amicon Model 8200 was used with Millipore membranes of regenerated cellulose of 10,000 NMWL (nominal molecular weight limit) by Amicon Technologies Pvt. Ltd. (Mumbai, India).

## 4. Conclusions

Core-shell gold and silver nanoparticles of different composition were successfully synthesized through a wet, reproducible multi-step procedure able to fabricate hybrid systems with tunable optical properties. The resulting nanostructures exhibit a heterogeneity degree in line with the best bottom-up methods based on chemical reduction in solution, although limitations in obtaining strict control of the products morphology were clearly highlighted. Employment of ω-amino-carboxylic acids of different chain lengths as protecting agents proved to represent an effective route to obtain stable products as well as to introduce additional surface functionalities by addressing the ligand self-assembly in a divergent fashion depending on the core nature. Further functionalization of the nanohybrids with selected fluorophores, namely the amino-derivatized benzofurazan BTEA for gold colloids and the carboxyl-substituted pyrene PyCA for silver colloids, was realized through a classic carbodiimide-assisted condensation, directly carried out in aqueous suspension. The dye-functionalized nanohybrids were characterized by photoluminescence quenching, the magnitude of which could be modulated by varying the covering degree of the fluorophore. This result opens the way to the potential use of this new class of core-shell nanostructures as fluorescent probes in sensing, optical or photonic platforms. Preliminary investigation on the filmability of nanohybrids/polymer mixtures enabled demonstration that the colloids can be successfully embedded in compatible polymeric matrix without causing undesirable segregation phenomena, also under severe experimental conditions. Nevertheless, poorly homogeneous films have been obtained so far by the dipping technique. Significant improvements on the film processing must be realized to exploit the best potential of these nanocomposite systems in all-polymer technological devices.

## Supplementary Materials

The following are available online at www.mdpi.com/1996-1944/9/12/997/s1. Figure S1: Experimental and calculated extinction spectra of 5APA@AuNPs and 8AOA@AuNPs in water; Figure S2: Experimental and calculated extinction spectra of aqueous suspensions of AuNPs and AgNPs protected with 5APA and 8AOA; Figure S3: UV-vis spectra of EDC-activated 5APA@AuNPs; Figure S4: Deconvolution of UV-vis spectrum of BTEA-5APA@AuNPs in water; Figure S5: UV-vis spectra of 8AOA@AuNPs embedded in PVAL and H-Chit/HyA matrices; Figure S6: Spectroscopic characterization of dye BTEA, Figure S7: Spectroscopic characterization of dye PyCA.
