# Supplementary material for: Synthesis of Fluorescent Core-Shell Metal Nanohybrids: A Versatile Approach
Source: Materials (Basel). 2016 Dec 9;9(12):997. doi: 10.3390/ma9120997 (PMC5457000; doi:10.3390/ma9120997)
Supplement: Supplementary file 1 [file materials-09-00997-s001.pdf]

Supplementary Materials: Synthesis of Fluorescent Core-Shell Metal Nanohybrids: A Versatile Approach

Marina Alloisio, Melania Rusu, Stefano Ottonello, Massimo Ottonelli, Sergio Thea and Davide Comoretto

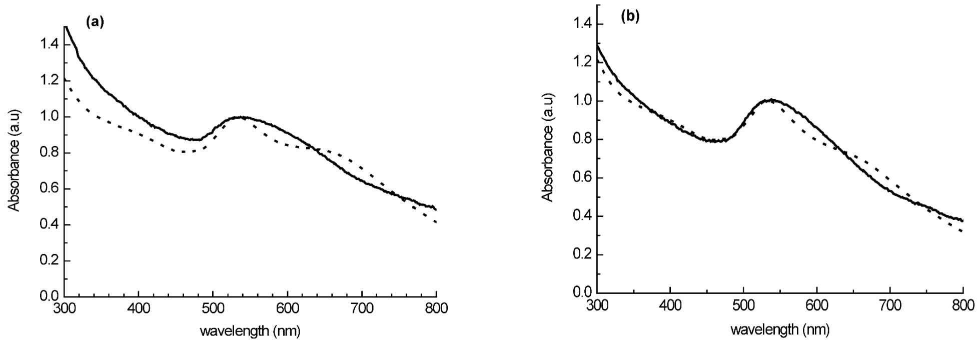

Figure S1. (**a**) Experimental (solid line) and calculated (dotted line) extinction spectra for an aqueous suspension of 5APA@AuNPs obtained by ligand-exchange reaction (LER) from PA@AuNPs template; (**b**) Experimental (solid line) and calculated (dotted line) extinction spectra for an aqueous suspension of 8AOA@AuNPs obtained by ligand-exchange reaction (LER) from PA@AuNPs template. Average fitting parameters: radius = 3.4 nm with σ = 0.11 nm; 33% spherical particles and 67% elongated particles with average aspect ratio = 2.2.

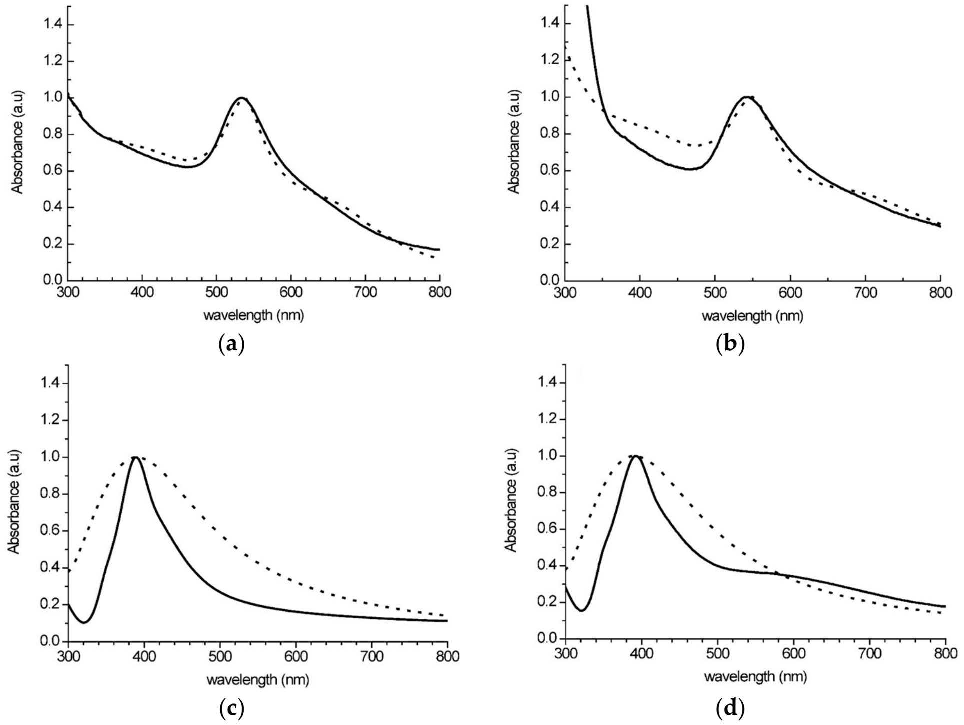

Figure S2. (**a**) Experimental (solid line) and calculated (dotted line) extinction spectra for an aqueous suspension of 5APA@AuNPs obtained from "naked" AuNPs template; (**b**) Experimental (solid line) and calculated (dotted line, blue) extinction spectra for an aqueous suspension of 8AOA@AuNPs from "naked" AuNPs template. Average fitting parameters: radius = 4.0 nm with σ = 0.85 nm; 89% spherical particles and 11% elongated particles with average aspect ratio = 1.7; (**c**) Experimental (solid line) and calculated (dotted line) extinction spectra for an aqueous suspension of 5APA@AgNPs obtained from "naked" AgNPs template; (**d**) Experimental (solid line) and calculated (dotted line) extinction spectra for an aqueous suspension of 8AOA@AgNPs from "naked" AgNPs template. Average fitting parameters: radius = 3.5 nm with σ = 0.05 nm; 98% spherical particles and 2% elongated particles with average aspect ratio = 2.0.

On the whole, the MG method was proved to be less effective in fitting experimental spectra of AgNPs, in particular in reproducing the profile features, owing to lower optimization in fitting parameters.

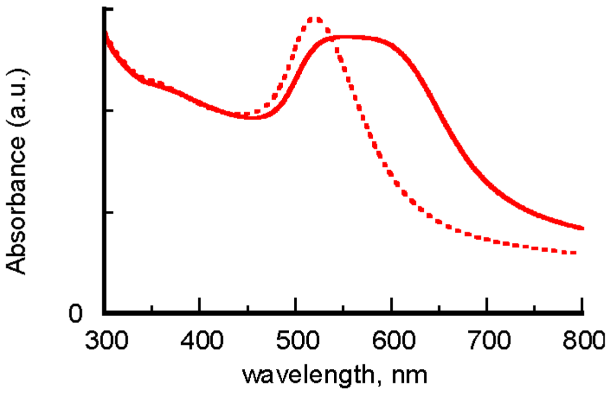

Figure S3. Absorption spectra of aqueous suspensions of 5APA@AuNPs before (dotted line) and after (solid line) being reacted with 1-(3-dimethylaminopropyl)-3-ethylcarbodiimide hydrochloride (EDC).

Separation of the Metal and Dye Contributions from the Spectrum of BTEA-5APA@AuNPs

In order to separate the contributions of the single components to the spectrum of BTEA-5APA@AuNPs, the following procedure was followed:

1. Assuming that the absorption properties of 2-(benzofurazan-4-thio)-ethylamine (BTEA) are not affected by the anchorage on the nanoparticle, the dye contribution was considered corresponding to the spectrum of free BTEA in water at the same concentration value of the bound dye, previously obtained from the purification procedure (5%);
2. The experimental UV-vis spectrum was subtracted point-to-point with that of the calculated dye component to obtain the contribution of the metal moiety;
3. The absence of artifacts or additional features in the spectral profile of the metal components (dashed line) confirms the quantitative determination of the anchored dye as well as the hypothesis that the nanohybrid spectrum is the sum of those of the single photoactive moieties.

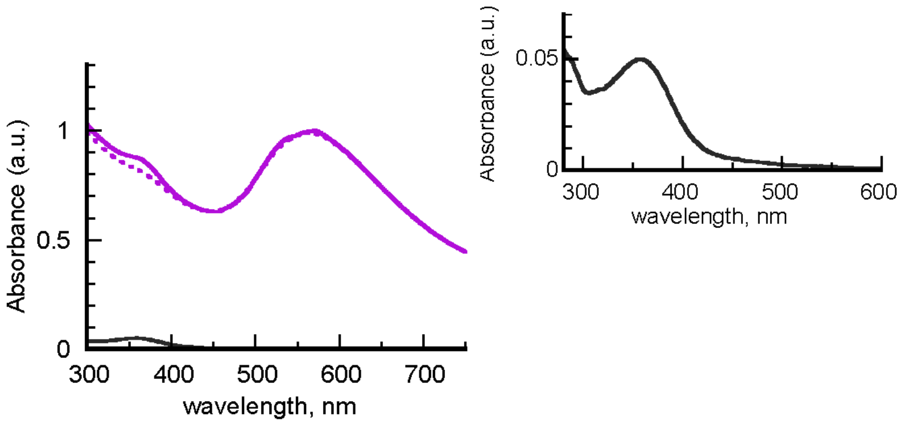

Figure S4. Absorption lineshapes of an aqueous suspensions of BTEA-5APA@AuNPs (solid purple line) and of the components BTEA (black solid line) and 5APA@AuNPs (purple dotted line). Inset: enlarged absorption spectrum of the BTEA component at the concentration value of bound dye.

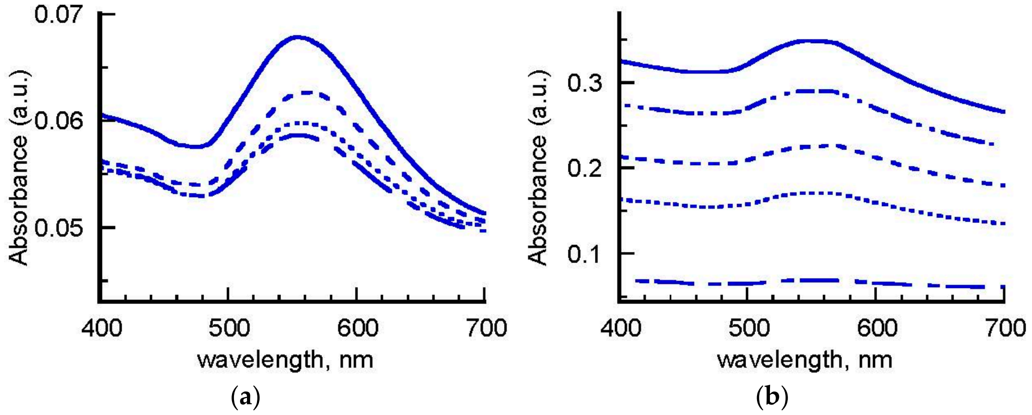

Figure S5. (**a**) Absorption spectra of multilayered films of 8AOA@AuNPs embedded in PVAL matrix, obtained by dipping technique; number of layers = 2 (dashed-dotted), 4 (dotted line), 6 (dashed line), 8 (solid line); (**b**) Absorption spectra of multilayered films of 8AOA@AuNPs embedded in chitosan matrix, obtained by alternate dipping with a hyaluronate matrix; number of layers = 2 (dashed-dotted line), 5 (dotted line), 8 (dashed line), 10 (double-dashed line), 12 (solid line).

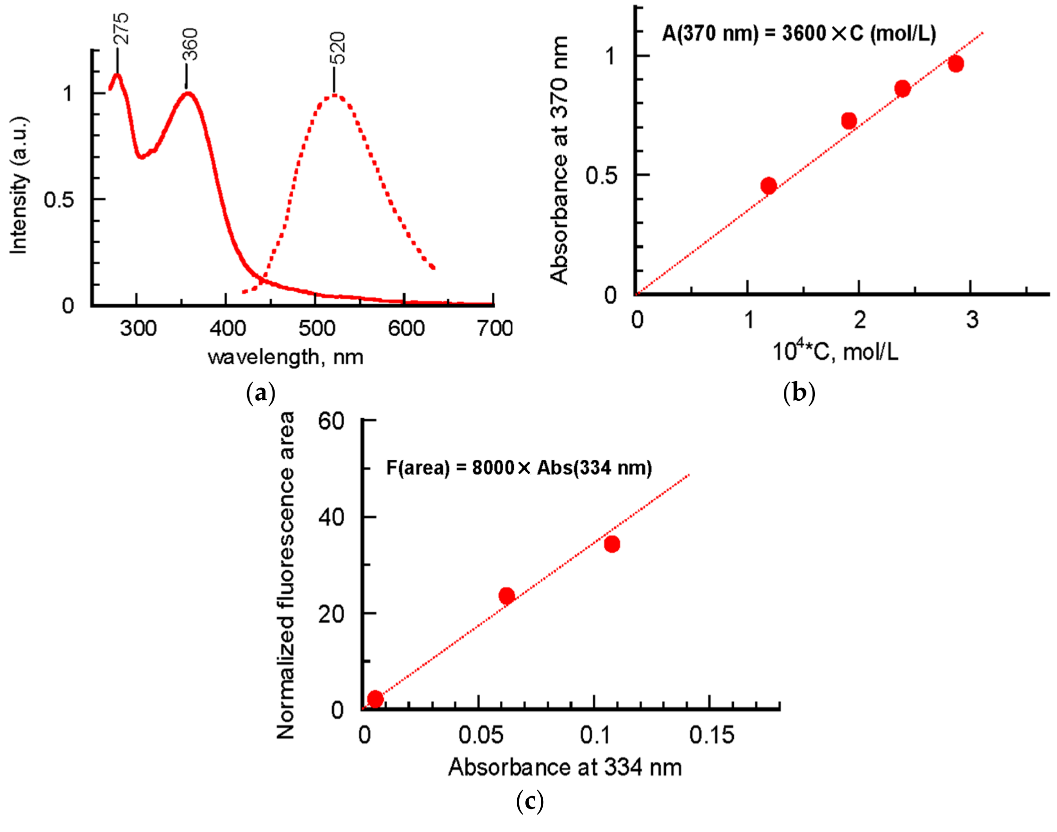

Figure S6. (**a**) Absorption (solid line) and fluorescence (dotted line) spectra of an aqueous solution of BTEA; excitation wavelength = 334 nm; (**b**) Titration curve of BTEA in water obtained by plotting absorbance at 370 nm vs. concentration (mol/L); (**c**) Titration curve of BTEA in water obtained by plotting normalized area of fluorescence vs. absorbance at 334 nm. Both titration curves were employed to determine the concentration of unbound dye from the wastewaters of purification.

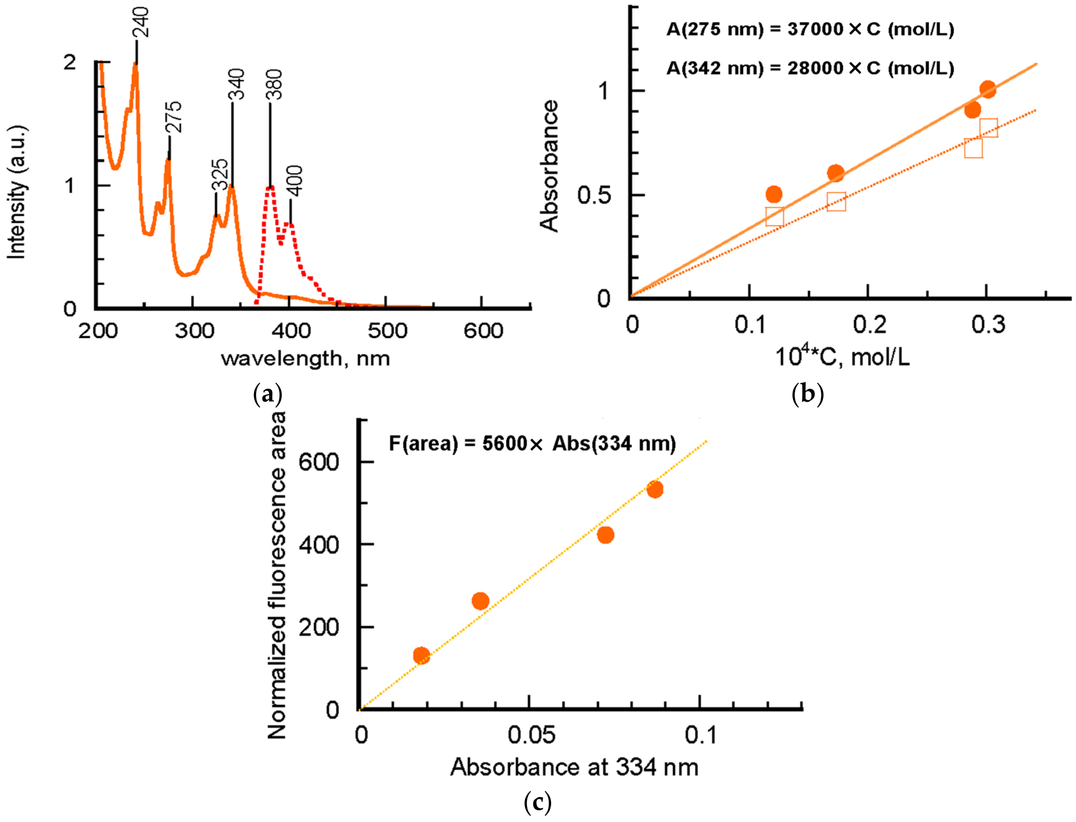

Figure S7. (**a**) Absorption (solid line) and fluorescence (dotted line) spectra of an aqueous solution of PyCA; excitation wavelength = 334 nm; (**b**) Titration curve of PyCA in water obtained by plotting absorbance at 275 nm and 342 nm vs. concentration (mol/L); (**c**) Titration curve of PyCA in water obtained by plotting normalized area of fluorescence vs. absorbance at 334 nm. Both titration curves were employed to determine the concentration of unbound dye from the wastewaters of purification.
